# The influence of trait anxiety on performance in the CatWalk test

**DOI:** 10.3389/fnbeh.2026.1682995

**Published:** 2026-04-28

**Authors:** Edoardo Parrella, Valentina Salari, Yasamin Meamarzadegan, Lorena Torroni, Vivekanand Jha, Giulia Spagnoli, Ali Jafari Fereidouni, Andrea Lazzarin, Vittoria Giuliari, Giuseppe Bertini, Paolo Francesco Fabene

**Affiliations:** 1 Departmental Faculty of Medicine, Saint Camillus International University of Health Sciences, Rome, Italy; 2 Section of Innovation Biomedicine, Department of Engineering for Innovation Medicine, University of Verona, Verona, Italy; 3 Section of Anatomy and Histology, Department of Neurosciences, Biomedicine and Movement Sciences, School of Medicine, University of Verona, Verona, Italy; 4 Unit of Epidemiology and Medical Statistics, Department of Diagnostics and Public Health, University of Verona, Verona, Italy

**Keywords:** anxiety, behavioral test, CatWalk, elevated plus maze (EPM), motor functions, mouse behavior

## Abstract

**Introduction:**

Trait anxiety refers to the individual variability in the predisposition to respond anxiously to stimuli. Anxiety has been shown to affect several physiological processes, including fine motor tasks, both in humans and in rodents. Therefore, trait anxiety may be a confounder factor in behavioral studies assessing motor functions in preclinical animal models. Among the tools employed to investigate motor functions in rodents, CatWalk XT is one of the most used. CatWalk XT is a computer-assisted apparatus that allows rapid and objective quantification of both static and dynamic gait parameters in rodents. The test consists of a training period in which the mouse learns to cross a platform and a test day in which the mouse footprints are recorded and analyzed.

**Methods:**

Here, we investigated whether trait anxiety, assessed with the Elevated Plus Maze (EPM) test, influences mice performance on CatWalk XT.

**Results:**

The results showed a correlation between anxiety levels as measured by the EPM and CatWalk test, with trait anxiety affecting the CatWalk learning and gait parameters. Indeed, mice with higher degrees of anxiety displayed a higher number of noncompliant runs during the training, ultimately protracting the time required by the experimenter to perform the CatWalk test. In addition, mice displaying fewer total entries in the EPM exhibited increased print lengths when assessed with the CatWalk system.

**Discussion:**

In conclusion, our findings indicate that individual differences in trait anxiety must be considered when testing mice with the CatWalk XT system. The use of specific anxiety tests before CatWalk testing may be useful to exclude those mice showing the highest levels of anxiety. This strategy would optimize researcher’s time, limit animals’ stress, and avoid errors in the results interpretation.

## 1 Introduction

Anxiety is a state of emotional and physical unease characterized by feelings of apprehension and tension, that arises from anticipation of a potential threat (Griffin, 1990; Hur et al., 2020; Samra et al., 2025). Anxiety can be divided into state and trait anxiety. While state anxiety reflects the transient response to a potential threat in a specific moment, trait anxiety refers to individual disposition to perceive the environment as threatening and is considered a trait of personality (Spielberger et al., 1983).

Individuals with higher levels of trait anxiety are more vulnerable to stress than those showing lower trait anxiety (Daviu et al., 2019). Anxiety has been shown to variously affect physiological processes in humans, including motor functions. Indeed, trait anxiety can impact motor performance when healthy people perform skilled activities that require attention in high-pressure contexts (Nieuwenhuys and Oudejans, 2012; Krasovsky et al., 2024). For example, high levels of anxiety lead musicians to perform imprecise movements during music performance (Kotani and Furuya, 2018).

The concept of trait anxiety originates from human psychology. In laboratory animals, we cannot directly measure psychological traits; rather, we infer trait-like anxiety on the basis of behavioral patterns obtained from standardized behavioral tests. However, evaluation of trait anxiety is relevant also in preclinical studies employing rodents, since certain animals may be prone to experience anxiety more frequently or intensely than others. Anxiety can heavily affect also rodents’ motor performance in behavioral tasks. Firstly, anxiety can impact on locomotor activity. In support of this, high anxiety-related behavior (HAB) rats, a selected rat strain characterized by high levels of trait anxiety, are less active than their low anxiety counterparts (Landgraf and Wigger, 2002). In addition, a recent study indicated that high anxiety traits may influence the control of skilled movements in facility-reared mice (Schiavo et al., 2023). Indeed, anxious mice showed extreme performance when tested with the ladder walking test, performing either really poorly or really effectively (Schiavo et al., 2023).

One of the most used systems worldwide to study motor functions in rodents is the CatWalk XT (Noldus Information Technology BV, Wageningen, The Netherlands). CatWalk XT is a computer-assisted system that allows a rapid and objective gait analysis in rodents (Garrick et al., 2021; Dickmann et al., 2022; Timotius et al., 2023). In the CatWalk XT apparatus rodents are filmed from beneath while crossing a glass plate, their footprints are recorded and analyzed by a software to generate a large number of gait parameters (Timotius et al., 2019). Although there is no universal gait parameter grouping, a general classification divides gait parameters into static and dynamic (Kappos et al., 2017). In recent years, the analysis of gait parameters obtained through the CatWalk XT has allowed the publication of several pre-clinical studies investigating neurological and movement disorders as well as injuries, including Parkinson’s disease (PD) (Witzig et al., 2023; Moceri et al., 2024; Izquierdo-Altarejos et al., 2024; Parrella et al., 2025), amyotrophic lateral sclerosis (ALS) (Guo et al., 2024), multiple sclerosis (Mohamed-Fathy Kamal et al., 2025), cerebellar ataxia (Suminaite et al., 2025), chorea-acanthocytosis (Riccardi et al., 2025), arthritis (Ritter et al., 2023), stroke (Salman et al., 2023; Mirzahosseini et al., 2024; Liu et al., 2024; Wang et al., 2024), traumatic brain injury (TBI) (Huo et al., 2023; Phuyal et al., 2025), spinal cord injury (SCI) (Liu et al., 2024; Su et al., 2024; Xing et al., 2024; Mojarad et al., 2025), peripheral nerve injury (Segelcke et al., 2023; Xian et al., 2023) [for less recent studies see the review (Timotius et al., 2023)].

Although alternative training methods have been investigated to reduce stress and anxiety in rodents subjected to the CatWalk test (Dickmann et al., 2022), to our knowledge the effect of trait anxiety on CatWalk task has never been analyzed. In the present study we analyzed the impact of anxiety on CatWalk performance of naïve NMRI male mice, a mouse strain commonly used in neurobehavioral research (Gower and Lamberty, 1993; Vicens et al., 1999; Griebel et al., 2000; Belzung et al., 2001; Yilmazer-Hanke et al., 2003; Kalueff and Tuohimaa, 2005; Petit-Demouliere et al., 2005; Gröticke et al., 2007; Gerami et al., 2024; Spagnoli et al., 2024). For this purpose, trait anxiety was evaluated in NMRI mice through the Elevated Plus Maze (EPM) test, a task that does not rely upon presentation of noxious stimuli and can therefore be considered an ethological way to value trait anxiety (Carobrez and Bertoglio, 2005). After being tested with the EPM test, NMRI mice were evaluated with the CatWalk XT apparatus, and the two behavioral performances were analyzed and compared.

## 2 Materials and methods

### 2.1 Experimental animals

The NMRI male mice, aged 5–7 months, used in this study were acquired from Envigo (Udine, Italy). The mice were housed in individually ventilated cages (IVCs), 2–4 animals per cage, with food and water *ad libitum*, and kept in a sound-attenuated room at constant temperature (22 ± 1.0°C) and humidity (60 ± 5%) with an inverted 12/12-h light-dark cycle with lights on at 7:00 p.m. Experimental procedures were conducted in accordance with the guidelines of the European Union directive 2010/63/EU and following the “Principles of Laboratory Animal Care” (NIH publication No. 86–23). All protocols were approved by the local ethics committee (C.I.R.S.A.L., University of Verona) and the Italian Ministry of Health.

### 2.2 Behavioral tests

**FIGURE 1:**
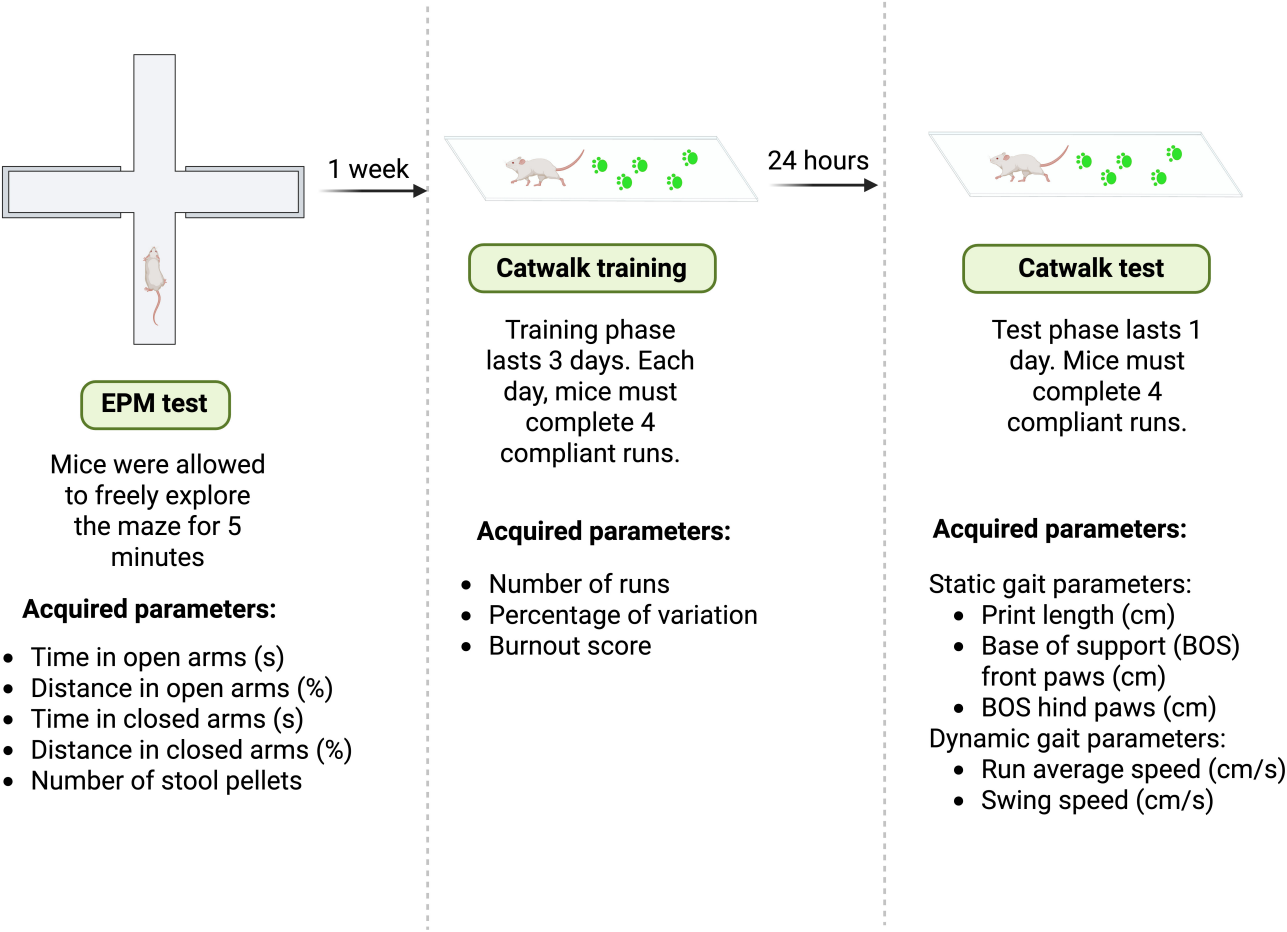
Experimental design. NMRI mice (*n* = 44) were tested for 5 min in the EPM to assess anxiety levels and were then returned to their home cages. The following parameters were recorded: time spent in the open arms (s), distance traveled in the open arms (%), time spent in the closed arms (s), distance traveled in the closed arms (%), and number of stool pellets. After 1 week, the mice were tested using the CatWalk system. The CatWalk test consists of 3 days of training followed by a fourth test day. During the training phase, mice were trained to traverse the CatWalk corridor without hesitation and while walking as straight as possible. To achieve this, on each training day the mice were required to complete four compliant runs. During the CatWalk training phase, the following parameters were recorded: the number of runs required to obtain four valid runs, percentage of variation, and burnout score. On the fourth day (test day), the gait performance of the mice was assessed as the average of four runs. Both static and dynamic gait parameters were evaluated. CatWalk static gait parameters: print length (cm), base of support (BOS) front paws (cm), BOS hind paws (cm). CatWalk dynamic gait parameters: run average speed (cm/s), swing speed (cm/s). Figure created with BioRender.com.

The NMRI male mice were tested with the EPM test, followed by the CatWalk XT test 1 week later. Mice were tested in all behavioral tasks consistently within the same time frame (10.00 a.m.–01.00 p.m.). The experimental design is shown in Figure 1.

#### 2.2.1 EPM test

The EPM test was used to measure anxiety behaviors (Pellow et al., 1985; di Nuzzo et al., 2015; Rangan et al., 2022). The EPM is one of the most widely used tests for assessing anxiety-like behavior and is based on the rodents’ natural aversion to open and elevated spaces, as well as on their spontaneous exploratory behavior in novel environments (Pellow et al., 1985; Walf and Frye, 2007; Komada et al., 2008). The maze consists of open arms and closed arms made of black opaque plastic (30 × 5 cm each), crossed in the middle perpendicularly to each other to form a center area (5 × 5 cm). The closed arms are surrounded by walls (15 cm height). During the test the mouse was placed onto the center area and allowed to freely explore the maze for 5 min and its behavior was recorded using an overhead video camera (HCD-3D800, Panasonic, Kadoma, Japan) and analyzed by the Ethovision XT software version 16 (Noldus Inc., Wageningen, The Netherlands). The apparatus was cleaned with ethanol 70% between each animal test.

During the task the following parameters were scored:

- Time in open arms (s): the total amount of time that the mouse spent exploring or remaining in the open arms.
- Distance in open arms (%): the percentage of distance moved in the open arms normalized to the total distance traveled.
- Time in closed arms (s): the total amount of time that the mouse spent exploring or remaining in the closed arms.
- Distance in closed arms (%): the percentage of distance moved in the closed arms normalized to the total distance traveled.
- Number of stool pellets.
- Total number of entries: the sum of entries into the closed and open arms.

Increased time and distance spent in the open arms are indicative of reduced anxiety-like behavior, whereas increased time and distance in the closed arms, along with a higher number of stool pellets, are associated with elevated anxiety levels (Komada et al., 2008; Burn et al., 2021; Rangan et al., 2022). The total number of entries is generally considered an index of overall locomotor activity and exploratory behavior; nevertheless, decreased total entries may also indicate increased anxiety-related freezing (Komada et al., 2008; Sestakova et al., 2013).

#### 2.2.2 CatWalk XT test

Approximately 1 week after EPM test, the mice were tested on the CatWalk XT 10.7 (Noldus Information Technology BV, Wageningen, The Netherlands). The CatWalk apparatus consists of a glass plate (130 × 20 × 0.5 cm) and a plastic corridor without floor and ceiling (120 × 5 cm) to narrow the running area on the glass plate. A movable lid equipped with a red-light lamp could cover the corridor providing a background illumination for video acquisition. At the end of the glass plate is mounted a plastic goal box where the mouse can hide at the end of the run. A high-speed color camera is placed below the glass plate. A source of LED green light is mounted alongside the long edge of glass plate and reflected inside the plate. When the mouse paws touch the surface of the glass plate, the green light is reflected 90° down and the signals of the paw area are captured by the camera and sent to the CatWalk XT software for analysis.

The test consisted of 3 days of training, and a fourth day in which gait parameters were recorded (test day) (Garrick et al., 2021). In the training period, the mouse was trained to cross the corridor without hesitation. Indeed, to be effectively analyzed, the mouse must walk at a constant speed with low variation and without changes of direction (Garrick et al., 2021; Dickmann et al., 2022). During the 3 days of training the mouse was subjected to a 1-h food restriction before the test. Then the animal was placed on one side of the corridor, the lid was closed, and the software registered a “run” when the mouse crossed the recording area, a selected virtual area slightly shorter than the corridor itself. During the recording period, the mouse was left to freely move in the corridor. Every time the mouse reached the goal box, it found a reward of highly palatable food and was left undisturbed to eat for 1 min. The recording was stopped automatically after four compliant runs were reached. At least three successful runs on the CatWalk are required to obtain reliable and representative gait parameters in rodents, as established by our laboratory and others (Baiguera et al., 2012; Timotius et al., 2023; Riccardi et al., 2025; Parrella et al., 2025). A run was considered non-compliant if it lasted < 1 s (the detection limit of the software) or more than 8 s, if the variation in walking speed exceeded 100%, or if the mouse changed direction, stopped moving, or reared against the walls of the corridor. Run validity parameters were determined according to both our laboratory experience and the CatWalk provider’s guidelines. The training allowed the animal to learn to traverse the corridor without hesitation and as straight as possible within a few seconds.

On the test day, the mouse was placed in the corridor as for the training period. No food restriction was implemented on the test day, although food reward was left in the goal box.

After testing each mouse, the equipment was accurately cleaned with 70% ethanol and dried with a paper towel.

During the 4 days in which the mice underwent the CatWalk task (3 days of training and the test day) we scored the Catwalk learning parameters, i.e., the parameters indicating the extent to which the mice have learned to perform the CatWalk task properly. The Catwalk learning parameters included:

- Number of runs: the total number of runs needed to obtain four compliant runs. The value was calculated as an average over 4 days of Catwalk task.
- Percentage of variation (% variation): it measures the variation in walking speed of the animal’s body. The value was calculated as an average over 4 days of Catwalk task.
- Burnout score: a score of “1” was assigned if, during a day of the CatWalk task, the number of runs was at least twice that of previous days, indicating burnout. A score of “0” was assigned if this condition was not met.

During the test day the CatWalk gait parameters were scored as average of four compliant runs.

CatWalk static gait parameters included:

- Print length (cm): the average length of the print areas of the four paws.
- Base of support (BOS) front paws (cm): the distance between the two front paws.
- BOS hind paws (cm): the distance between the two hind paws.

CatWalk dynamic gait parameters included:

- Run average speed (cm/s): walk speed measured as distance over time.
- Swing speed (cm/s): the average speed of the four paws during the swing phase, i.e., the period when the paw is not in contact with the glass plate.

### 2.3 Statistical analysis

The sample included 44 mice, which was the total number of animals available for this study. To assess the sensitivity of the analysis, a *post-hoc* power calculation was conducted using G*Power (version 3.1.9.7). The analysis was based on a significance level of α = 0.05, a null hypothesis correlation of ρ = 0, and an alternative hypothesis correlation of ρ = 0.45, reflecting a moderate effect size commonly observed in behavioral studies. Results indicated that with *n* = 44, the study had a statistical power of > 80% to detect a correlation of this magnitude. This estimate is provided to contextualize the detectable effect size given the available sample, rather than as a justification for sample size determination.

Prior to analyses, the normality of data distributions was assessed using the D’Agostino-Pearson omnibus test for each variable and within each group when relevant.

The primary analysis aimed to evaluate the relationship between anxiety-related behavioral parameters obtained from the EPM and the parameters measured by the CatWalk system in the same mice. Depending on the data distribution, Pearson’s correlation coefficient (for normally distributed and linearly related variables) or Spearman’s rank correlation coefficient (for non-normally distributed or ordinal variables) was used to quantify associations. To assess the relationship between continuous predictors and the dichotomous variable (burnout score), a binary logistic regression was performed. In this case results were reported as odds ratios (OR) with 95% confidence intervals (CI), along with *p*-values.

As a secondary analysis, to assess the association between anxiety levels and CatWalk parameters, animals were categorized into three groups representing low, medium and high anxiety levels (LA, MA and HA, respectively), based on tertile splits of each of the following behavioral indices: (i) time in open arms, (ii) distance in open arms, (iii) time in closed arms, (iv) distance in closed arms, (v) number of stool pellets, (vi) total number of entries. This subdivision generated three independent groupings of the same animal cohort, one for each behavioral index. Each classification was used to compare the groups in the following parameters: number of runs, % variation, burnout score, print length, BOS of the front and hind paws, run average speed, and swing speed. For comparisons between anxiety-based groups (LA, MA, HA), a one-way ANOVA was used, followed by Tukey’s *post-hoc* test when the assumption of normality and homogeneity of variances were met. For non-normally distributed data, the Kruskal-Wallis test was applied, followed by Dunn’s *post-hoc* test for multiple comparisons. All tests were two-tailed, and the significance level was set at *p* < 0.05. When multiple variables were tested across groups, correction for multiple comparisons was applied using either the Bonferroni or False Discovery Rate (FDR) method, as appropriate. Results are reported as mean ± standard deviation (SD) or median and interquartile range (IQR), depending on the data distribution.

All analyses were conducted using GraphPad Prism version 10.0 for Windows (GraphPad Software, San Diego, CA, United States) on complete datasets, and no samples were excluded arbitrarily.

## 3 Results

### 3.1 Correlation between EPM anxiety parameters and CatWalk learning parameters

**FIGURE 2:**
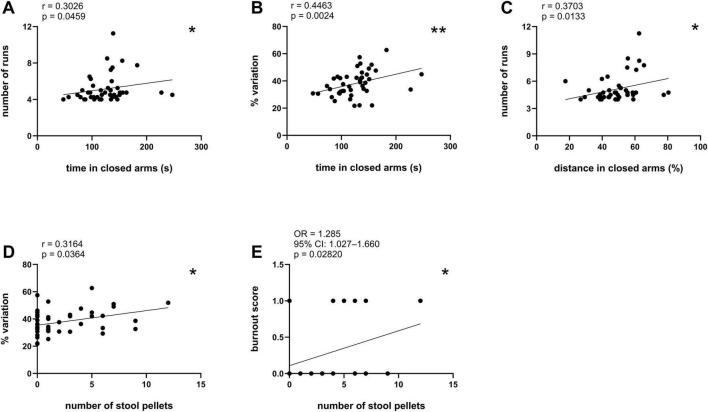
Significant correlations between EPM anxiety parameters and CatWalk learning parameters. The following correlations are shown: **(A)** time in closed arms (s) and number of runs, *p < 0.05; **(B)** time in closed arms (s) and% variation, **p < 0.01; **(C)** distance in closed arms (s) and number of runs, *p < 0.05; **(D)** number of stool pellets and% variation, *p < 0.05; **(E)** number of stool pellets and burnout score, *p < 0.05. Spearman correlation in **(A–C)**, Pearson correlation coefficients in **(D)**, binary logistic regression in **(E)**.

Approximately 1 week after being tested on the EPM, the mice performed the CatWalk test. The CatWalk test consisted of 3 days of training, in which the mouse learned to move from one side of the corridor to the other without hesitation, followed by 1 day of testing. First, we analyzed the correlation between the anxiety-related parameters scored with the EPM test (time in open arms, distance in open arms, time in closed arms, distance in closed arms, number of stool pellets, total number of entries) and CatWalk learning parameters (number of runs, % variation, burnout score) (Table 1). Among all tested associations, significant positive correlations were found between time in closed arms and number of runs (Figure 2A, *r* = 0.3026, *p < 0.05, Spearman correlation), between time in closed arms and % variation (Figure 2B, *r* = 0.4463, **p < 0.01, Spearman correlation), between distance in closed arms and number of runs (Figure 2C, *r* = 0.3703, *p < 0.05, Spearman correlation), between the number of stool pellets and% variation (Figure 2D, *r* = 0.3186, *p < 0.05, Pearson correlation coefficients), and between the number of stool pellets and burnout score as assessed by binary logistic regression (Figure 2E, OR = 1.285, 95% CI: 1.027–1.660, *p < 0.05, binary logistic regression). No significant correlation was found between time and distance in the open arms, total number of entries, and CatWalk learning parameters.

**TABLE 1:** Correlation between EPM anxiety parameters and CatWalk learning parameters.

EPM anxiety parameter	Number of runs	% Variation	Burnout score
Time in open arms	*r* = –0.1827; *p* = 0.2352	*r* = –0.2175; *p* = 0.1561	OR = 0.9924 95% CI: 0.9708–1.011 *p* = 0.4435
Distance in open arms	*r* = –0.2277; *p* = 0.1371	*r* = –0.2038; *p* = 0.1845	OR = 0.9594 95% CI: 0.8896–1.021 *p* = 0.1980
Time in closed arms	*r* = 0.3026; *p = 0.0459	*r* = 0.4463; **p = 0.0024	OR = 1.011 95% CI: 0.9923–1.031 *p* = 0.2481
Distance in closed arms	*r* = 0.3703; *p = 0.0133	*r* = 0.2828; *p* = 0.0629	OR = 1.021 95% CI: 0.9617–1.087 *p* = 0.5006
Number of stool pellets	*r* = 0.2429; *p* = 0.1048	*r* = 0.3186; *p = 0.0351	**OR = 1.285** **95% CI: 1.027–1.660** *p = 0.02820
Total number of entries	*r* = 0.1413; *p* = 0.3601	*r* = –0.01269; *p* = 0.9349	OR = 1.021 95% CI: 0.961–1.085 *p* = 0.504

The correlation coefficients (r) with corresponding *p*-values, or odds ratios (OR) with 95% confidence intervals (CI) and *p*-values, are reported. Statistically significant correlations (*p* < 0.05) are indicated in bold. *p < 0.05, **p < 0.01.

**FIGURE 3:**
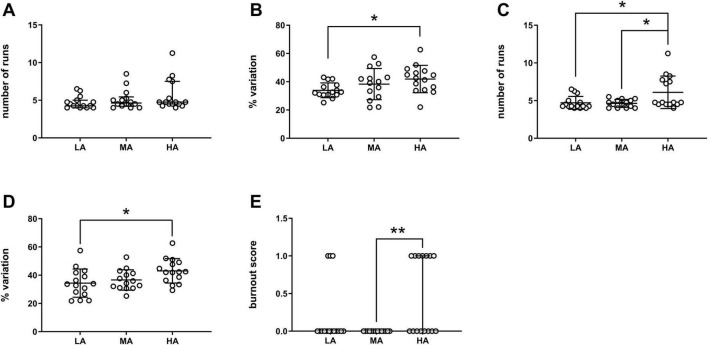
CatWalk learning parameters grouped according to anxiety levels. The CatWalk learning parameters were plotted according to the groups determined by the EPM parameters for which a significant correlation was found: **(A)** number of runs grouped by the time spent in closed arms; **(B)** % variation grouped by the time spent in closed arms; **(C)** number of runs grouped by the distance in closed arms; **(D)** % variation grouped by the number of stool pellets; **(E)** burnout score grouped by the number of stool pellets. LA (low anxiety): 15 mice; MA (medium anxiety): 14 mice; HA (high anxiety): 15 mice. *p < 0.05, **p < 0.01; Kruskal-Wallis followed by Dunn’s multiple comparisons test in **(A,E)**, One-Way ANOVA followed by Tukey’s multiple comparisons test in (**B–D)**. Data are expressed as median ± IQR in **(A,E)**, and as mean ± SD in (**B–D)**.

To further explore the association between anxiety levels and CatWalk learning parameters, animals were divided into three groups (LA, MA, and HA) based on tertiles calculated separately for (i) time in closed arms, (ii) distance in closed arms, and (iii) the number of stool pellets produced during the test. This grouping approach supported our correlational findings. Specifically, when grouping was based on time spent in the closed arms, the HA group showed a significantly greater % variation than the LA group (Figure 3B: LA vs. HA, *p < 0.05, One-Way ANOVA followed by Tukey’s multiple comparisons test). When groups were defined by distance traveled in the closed arms, the HA group had a significantly higher number of runs than both the LA and MA groups (Figure 3C: LA vs. HA, *p < 0.05; MA vs. HA, *p < 0.05, One-Way ANOVA followed by Tukey’s multiple comparisons test). Similarly, when grouping was based on the number of stool pellets, the HA group showed a significantly greater% variation compared to the LA group (Figure 3D: LA vs. HA, *p < 0.05, One-Way ANOVA followed by Tukey’s multiple comparisons test), and a significantly higher burnout score compared to the MA group (Figure 3E: MA vs. HA, **p < 0.01, Kruskal-Wallis followed by Dunn’s multiple comparisons test).

Although no statistically significant differences in the number of runs were observed across groups based on time in closed arms, a progressive increase was apparent (Figure 3A, *p* > 0.05, Kruskal-Wallis followed by Dunn’s multiple comparisons test; mean number of runs: LA = 4.7, MA = 5.1, HA = 5.7).

### 3.2 Correlation between EPM anxiety parameters and CatWalk static gait parameters

**FIGURE 4:**
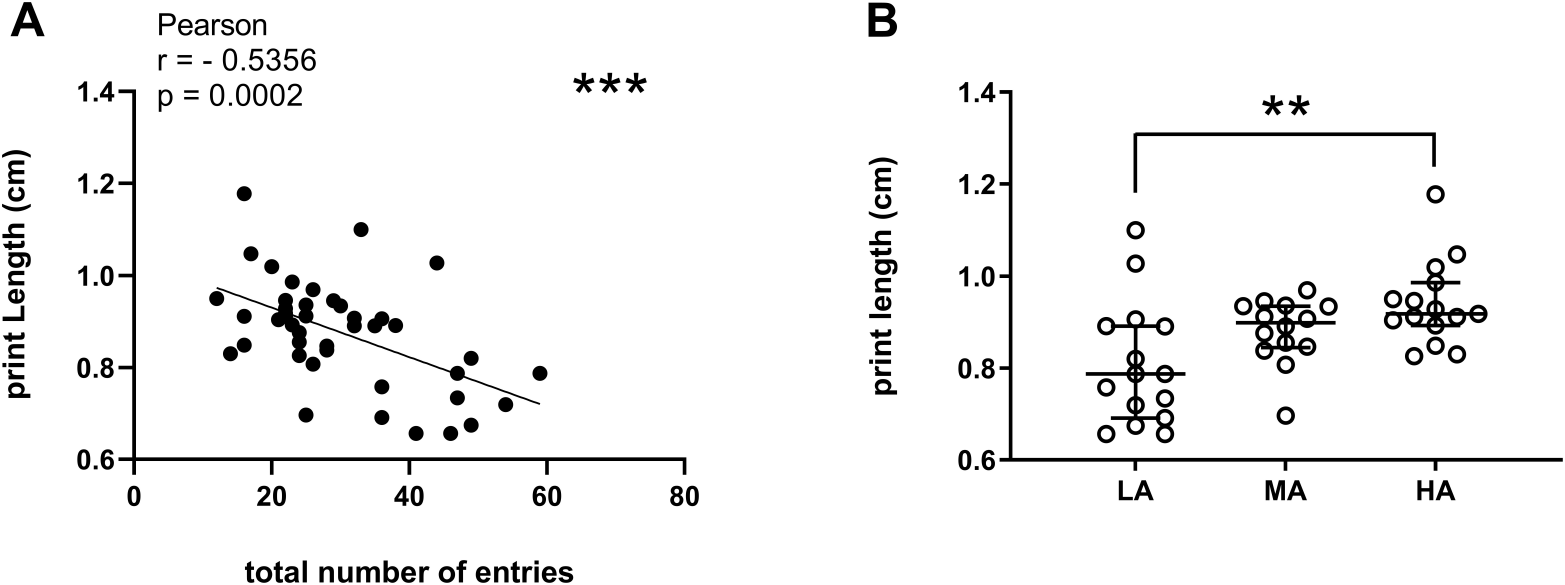
**(A)** Significant correlation between EPM total number of entries and the CatWalk static gait parameter print length (cm). ***p < 0.001, Pearson correlation coefficients. **(B)** CatWalk print length (cm) grouped by EPM total number of entries. LA (low anxiety): 15 mice; MA (medium anxiety): 14 mice; HA (high anxiety): 15 mice. **p < 0.01; Kruskal-Wallis followed by Dunn’s multiple comparisons test. Data are expressed as median ± IQR.

On the test day, both static and dynamic gait parameters were evaluated as average of four runs without hesitation. First, we investigated the correlation between the anxiety-related parameters scored with the EPM test and CatWalk static gait parameters (print length, BOS of front paws, BOS of hind paws) (Table 2). Total entries in the EPM were significantly negatively correlated with print length measured in the CatWalk (Figure 4A, *r* = –0.5356; ***p < 0.001, Pearson correlation coefficients). No significant correlations were found between the other EPM anxiety-related measures and the static gait parameters.

**TABLE 2:** Correlation between EPM anxiety parameters and CatWalk static gait parameters.

EPM anxiety parameter	Print length	BOS front paws	BOS hind paws
Time in open arms	*r* = –0.08893; *p* = 0.5659	*r* = 0.03539; *p* = 0.8196	*r* = –0.2510; *p* = 0.1003
Distance in open arms	*r* = –0.1696; *p* = 0.2711	*r* = 0.07976; *p* = 0.6068	*r* = –0.2093; *p* = 0.1726
Time in closed arms	*r* = –0.01198; *p* = 0.9385	*r* = 0.2222; *p* = 0.1471	*r* = 0.2732; *p* = 0.0728
Distance in closed arms	*r* = –0.1299; *p* = 0.4005	*r* = 0.08966; *p* = 0.5627	*r* = 0.2234; *p* = 0.1290
Number of stool pellets	*r* = 0.1210; *p* = 0.4341	*r* = 0.05215; *p* = 0.7367	*r* = 0.09696; *p* = 0.5317
Total number of entries	***r* = –0.5356;** ***p = 0.0002	*r* = 0.05227; *p* = 0.7361	*r* = –0.1303; *p* = 0.3992

Correlation coefficients (r) and their associated *p*-values are reported. Statistically significant correlations (*p* < 0.05) are indicated in bold. ***p < 0.001.

To further investigate this association, we compared print length values across groups stratified according to the total number of entries. The results of this analysis corroborated the observed correlation, as the HA group exhibited significantly greater print length compared to the LA group (Figure 4B: LA vs. HA, **p < 0.01, Kruskal-Wallis followed by Dunn’s multiple comparisons test).

### 3.3 Correlation between EPM anxiety parameters and CatWalk dynamic gait parameters

**FIGURE 5:**
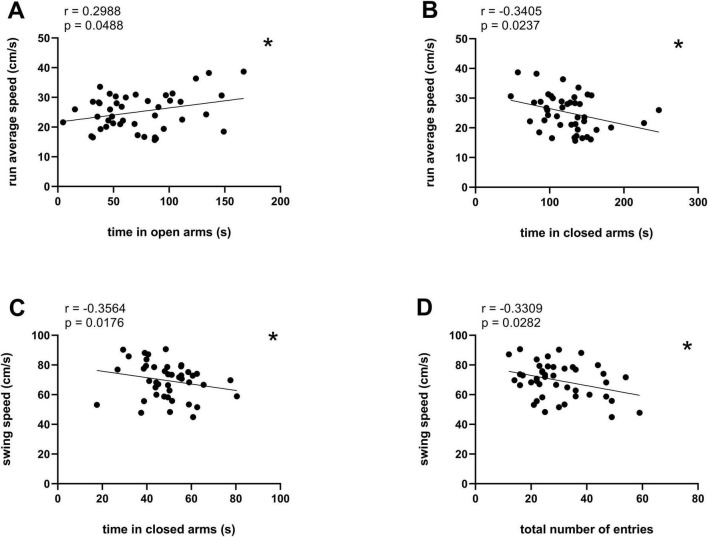
Significant correlations between EPM anxiety parameters and CatWalk dynamic gait parameters The following correlations are shown: **(A)** Time in open arms (s) and run average speed (cm/s), *p < 0.05; **(B)** time in closed arms (s) and run average speed (cm/s), *p < 0.05; **(C)** time in closed arms (s) and swing speed (cm/s), *p < 0.05. **(D)** Total number of entries and swing speed (cm/s), *p < 0.05. Pearson correlation coefficients in **(A,D)**, Spearman correlation in **(B,C)**.

Finally, we analyzed the correlation between the anxiety-related behavior in the EPM and CatWalk dynamic gait parameters, specifically run average speed and swing speed (Table 3). The analysis revealed a significant positive correlation between time in open arms and run average speed (Figure 5A, *r* = 0.2988, *p < 0.05, Pearson correlation coefficients). In contrast, time spent in the closed arms correlated negatively with both average speed (Figure 5B, *r* = –0.3405, *p < 0.05, Spearman correlation) and swing speed (Figure 5C, *r* = –0.3564, *p < 0.05, Spearman correlation), and total number of entries correlated negatively with swing speed (Figure 5D, *r* = –0.3309, *p < 0.05, Pearson correlation coefficients).

**TABLE 3:** Correlation between EPM anxiety parameters and CatWalk dynamic gait parameters.

EPM anxiety parameter	Run average speed	Swing speed
Time in open arms	***r* = 0.2988;** *p = 0.0488	*r* = 0.1607; *p* = 0.2975
Distance in open arms	*r* = 0.2541; *p* = 0.0960	*r* = 0.05095; *p* = 0.7426
Time in closed arms	***r* = –0.3405;** *p = 0.0237	***r* = –0.3564;** *p = 0.0176
Distance in closed arms	*r* = –0.2030; *p* = 0.1863	*r* = –0.2198; *p* = 0.1517
Number of stool pellets	*r* = –0.1565; *p* = 0.3102	*r* = –0.1718; *p* = 0.2649
Total number of entries	*r* = 0.02939; *p* = 0.8498	***r* = –0.3309;** *p = 0.0282

Correlation coefficients (r) and their associated *p*-values are reported. Statistically significant correlations (*p* < 0.05) are indicated in bold. *p < 0.05.

**FIGURE 6:**
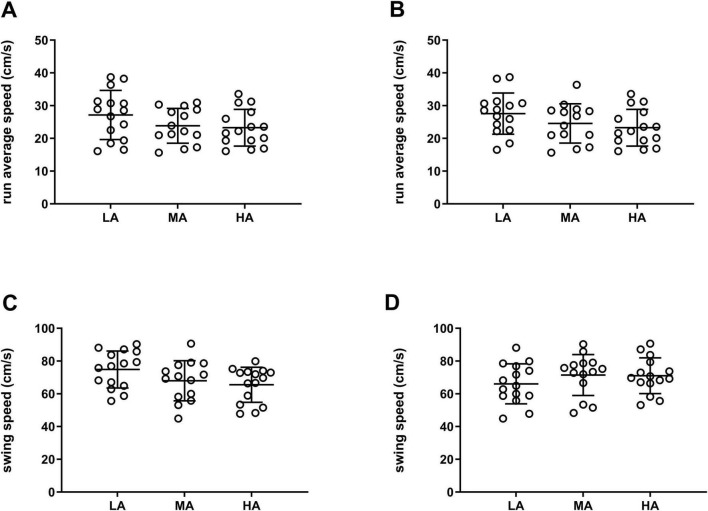
CatWalk dynamic gait parameters grouped based on anxiety levels. The CatWalk dynamic gait parameters were plotted according to the groups determined by the EPM parameters for which a significant correlation was found: **(A)** Run average speed (cm/s) grouped by the time spent in open arms; **(B)** run average speed (cm/s) grouped by the time spent in closed arms; **(C)** swing speed (cm/s) grouped by the time spent in closed arms. **(D)** Swing speed (cm/s) grouped by the total number of entries. LA (low anxiety): 15 mice; MA (medium anxiety): 14 mice; HA (high anxiety): 15 mice. *p* > 0.05; One-Way ANOVA followed by Tukey’s multiple comparisons test. Data are expressed as mean ± SD.

To further examine these associations, we compared the average running speed across groups defined by time spent in the open arms and time spent in the closed arms (Figures 6A,B, respectively); we also compared swing speed across groups defined by time spent in the closed arms and total number of entries (Figures 6C,D, respectively). However, no significant differences between the anxiety-level groups (LA, MA, and HA) were observed in any of the parameters (*p* > 0.05, One-Way ANOVA followed by Tukey’s multiple comparisons test).

## 4 Discussion

Our findings demonstrate a significant association between anxiety level in naïve mice and performance in the CatWalk test.

Behavioral data from the EPM confirmed a wide interindividual variability in anxiety traits, in line with previous reports in rodents (Ho et al., 2002; Kobayashi et al., 2021).

Anxiety affects the CatWalk learning parameters. Indeed, we observed a positive correlation between the time spent in the EPM closed arms and the number of runs and % variation measured during the CatWalk task, between the distance in the EPM closed arms and CatWalk number of runs, as well as between the number of stool pellets produced in the EPM and CatWalk % variation and burnout score. In support of this, mice classified by the time spent in closed arms, the distance in closed arms and the number of stool pellets as displaying an elevated anxiety proneness (HA mice), showed higher number of runs, % variation and burnout score than mice showing lower anxiety levels (MA or LA mice).

A correlation was also observed between EPM measures and CatWalk static parameters. Specifically, we found a significant negative correlation between the total number of arm entries and print length, which was further confirmed by the analysis of the stratified print length values. Mice with lower total entries, potentially reflecting increased anxiety-related freezing (Komada et al., 2008; Sestakova et al., 2013), exhibited longer paw prints. It can be speculated that more anxious mice may move less in the EPM and adopt a more cautious walking style on the CatWalk, resulting in longer paw prints as they place their paws more fully for stability. Future analyses are needed to further clarify the significance of this correlation.

A more modest association was observed between anxiety measures and dynamic gait parameters assessed on the test day. Indeed, we observed a correlation between the parameters scored with the EPM test (time in the open arms, time in the closed arms, and total number of entries) and the dynamic gait parameters measured with the CatWalk (run average speed and swing speed). The correlation was positive for the time in open arms, and negative for the time in closed arms and total number of entries, suggesting that anxious mice could move slower in the CatWalk apparatus. Notably, the reduction in run and swing speed is in line with the previously observed increase in print length. However, we did not find any significant difference between the mice groups classified by anxiety levels. This discrepancy may reflect within-group variability or limited power to detect small effects, underscoring the necessity for further investigations with larger sample cohorts.

Different studies pointed out a correlation between anxiety and motor activity in rodents (Metz et al., 2001; Martins et al., 2024). For example, HAB mice exhibit reduced locomotor activity in stressful contexts compared with normal-anxiety counterparts (Gaburro et al., 2011). When tested in the open field or in the EPM, HAB rats were less active and presented decreased locomotor activity than low anxiety rats (Liebsch et al., 1998; Landgraf and Wigger, 2002). Anxiety may influence not only exploratory and locomotor activity but also motor skills. It has been shown that skilled walking performance can be influenced by an aversive and extinction-resistant experience in rats (Medeiros et al., 2018), or by anxiety traits in mice (Schiavo et al., 2023). Notably, in accordance with our findings, a recent research on CatWalk XT test demonstrated that stressed untrained mice needed a higher number of runs to complete the task and displayed a lower running speed than less anxious trained animals (Dickmann et al., 2022).

This study presents some limitations. First and foremost, it is important to consider that NMRI mice are among the mouse strains displaying low anxiety levels (Vicens et al., 1999; Griebel et al., 2000; Belzung et al., 2001; Kalueff and Tuohimaa, 2005). Notably, despite this trait, we observed clear correlations between anxiety-related behaviors and CatWalk performance. This suggests that in strains with inherently higher anxiety, the impact on learning and CatWalk parameters may be even more pronounced. Follow-up studies using more anxiety-prone strains could help to validate and extend these findings.

Second, in this study we employed only male mice. Recent publications indicate sex as one of the factors affecting gait performance assessed by CatWalk (Pitzer et al., 2021; Dickmann et al., 2022). The sex-related differences observed in the CatWalk test may be due to intrinsic differences in heart and skeletal muscle, or to hormonal status (Kadi et al., 2002; Konhilas et al., 2004). Moreover, sex hormones influence cognition and response to stress (Milad et al., 2009; ter Horst et al., 2012), and EPM results (Kobayashi et al., 2021). Therefore, further studies are needed to investigate the correlation between anxiety and CatWalk test also in female rodents.

## 5 Conclusion

Our results indicate that individual differences in trait anxiety need to be taken into account when testing mice with the CatWalk XT system.

Anxiety levels impact on mice learning, potentially increasing the number of non-compliant runs and animals failing to meet run criteria, and ultimately protracting the time required by the experimenter to perform the CatWalk test.

Importantly, anxiety may interfere also with CatWalk gait parameters, increasing the print length and reducing the walk and swing speed of the tested mice. Although further experiments are required to validate this hypothesis, these findings should be considered especially when testing mice modeling diseases in which these parameters are altered. Indeed, CatWalk run speed is always decreased in stroke models, while swing speed is always reduced in stroke, SCI, and PD models (Timotius et al., 2023).

In light of these considerations, the use of protocols designed to reduce animals’ stress during handling and CatWalk testing is highly recommended to minimize interference from trait anxiety (Dickmann et al., 2022). Moreover, the use of one or more anxiety behavior tests (such as EPM) before CatWalk testing may be useful to identify and exclude those mice showing the highest levels of anxiety. This strategy would optimize the experimenter’s time and improve mice performance in the CatWalk test. In addition, the exclusion of anxious animals would avoid them additional stress during the CatWalk test, fulfilling the refinement principle of the 3Rs principles (replace, reduce, and refine) for the ethical use of animals in scientific research.

## Data Availability

The raw data supporting the conclusions of this article will be made available by the authors, without undue reservation.
